# Effect of Multi-Walled Carbon Nanotubes on Improving the Toughness of Reactive Powder Concrete

**DOI:** 10.3390/ma12162625

**Published:** 2019-08-17

**Authors:** Jintao Liu, Hang Jin, Xin Zhao, Cheng Wang

**Affiliations:** 1 College of Civil Engineering and Architecture, Zhejiang University of Technology, Hangzhou 310014, China; 2 School of Civil Engineering and Architecture, Zhejiang University of Science and Technology, Hangzhou 310058, China; 3 College of Water Resources and Architectural Engineering, Tarim University, Alear, Xinjiang 843300, China

**Keywords:** carbon nanotubes, microstructure, tensile strength, mechanical properties

## Abstract

Multi-walled carbon nanotubes (MWCNTs) have great potential to improve the strength and microstructure of traditional cement-based materials. In this research, different aspect ratios of MWCNTs (F-type and L-type) were dispersed into water using surfactants, and then incorporated into reactive powder concrete (RPC) for improving mechanical and microstructure properties. With the addition of 0.025 wt.% F-MWCNTs, the 28 days compressive strength and initial-cracking flexural strength increased by 7.2% and 36%, respectively. Moreover, the first-cracking tensile strengths of the composites containing L-MWCNTs were improved by 16%. Energy absorption capability indices were formulated based on tensile load–displacement curves, and results showed that the energy absorption capabilities of RPC at initial cracking improved as a result of the incorporation of MWCNTs. Furthermore, microscopic analysis indicated that MWCNTs decelerate crack development at the nanoscale and improve the initial-cracking tensile strength of RPC.

## 1. Introduction

Reactive powder concrete (RPC) with extremely high strength and durability was developed by Pierre at the end of the last century and has been widely used in long-span bridges, concrete penstock and containment structures due to its excellent performance [1]. The fracture energy of RPC is up to 40000 J/m^2^, which is 250 times that of ordinary concrete [2]. In addition, RPC can prevent effectively the penetration of chloride ion and exhibits excellent durability, which benefit from its dense microstructure [3]. At present, the tensile strength of RPC is only about a tenth to a twentieth of the compressive strength, and it needs to be further improved [4]. According to Zheng et al. [5], the tensile strength of RPC without steel-fiber reinforcement was only about 5.7 MPa, while it was 8.6 MPa when a 2 vf.% of steel-fiber was added. In another study, the dynamic tensile behavior of reactive powder concrete was investigated. Results showed that the static tensile strength of RPC was 5.0 MPa, and RPC’s dynamic tensile stress rapidly increased with an increased strain rate [6]. Furthermore, Benjamin and Florent investigated the influence of steel fiber volume fraction on the uniaxial tensile mechanical response of RPC, and the results revealed that the cracking strength of RPC with 2 vf.% and 2.5 vf.% steel fiber were 8.5 and 8.4 MPa, respectively. [7]. Although RPC has a highly dense matrix, the formation of internal nano voids and micro-cracks inevitably affect the cracking strength of RPC [8,9]. Meanwhile, traditional fiber can only restrain the macrocrack propagation, but has failed to prevent the development of microcrack and reduced micro-defects of RPC [10,11].

RPC is a multiphase cement composite, and its mechanical performance is affected mainly by matrix strength, fiber dosage, fiber aspect ratio, curing regime, etc. Generally, the compressive strength of RPC increased with the increasing matrix density and strength, and the fiber–matrix bond strength also improved. With proper fibers, the post cracking strength, strain capacity, and multiple micro-cracking behavior of RPC could be improved significantly [12,13]. Recently, carbon nanotubes (CNTs) were considered promising nano fibers for improving microstructure of cementitious materials [14]. The tensile strength of CNTs could reach 63 GPa, and its yield strain is greater than 10% [15,16]. In addition, the aspect ratios of CNTs was approximately 1000 in general, which was much higher than traditional fibers [17]. Konsta-Gdoutos et al. [18,19] investigated the influence of different lengths and concentrations of MWCNTs on the mechanical properties of cement pastes, and found that 0.048 wt.% long MWCNTs and 0.08 wt.% short MWCNTs raised the flexural strength by 25%. Li et al. [20] used surface-treated MWCNTs to improve the interfacial strength between CNTs and the hydrations of cement, and results showed that chemical reactions took place between the carboxylic acid groups and the calcium silicate hydrate (C–S–H) or Ca(OH)_2_. Cwirzen et al. [21] studied the ductility of cement paste with different modified MWCNTs, and results showed that the compressive strength of samples increased by 50% with the addition of 0·045–0·15 wt.% MWCNTs regarding the control. Meanwhile, researchers also reported that MWCNTs provided larger interface for stress transfer and limited the growth of nano-size cracks, and hindered the formation of micro-size pores [14,22,23,24]. As nano reinforcement, MWCNTs tend to form aggregates due to the high aspect ratios and van der Waals, and these aggregates might emerge later as matrix defects in cement composites [25,26]. Therefore, different kinds of superficial active agents and an ultrasonic dispersion method were applied to solve the agglomeration problem of MWCNTs [27,28,29]. Although MWCNTs have extremely high tensile strength, the surface of MWCNTs was inert and hard to react with hydration products. Thus, improving the bond strength between MWCNTs and hydration products was another crucial factor. MWCNTs could provide a stronger interface for stress transfer and delay micro-crack propagation with a high bond force [30,31].

However, most of the previous studies have focused on the CNTs reinforcing cement paste or mortar, and little research has been carried out on the CNTs modification of RPC. The objective of this research was to study the effect of different types of MWCNTs on the microstructure and mechanical properties of RPCs. Different concentrations of MWCNTs (0.025%, 0.05%, 0.1%, and 0.2% by weight of cement) were added to the RPC. The compressive strength, flexural strength and direct tensile strength of composites were evaluated. Furthermore, a toughness characterization method was proposed to reveal the energy absorption capability of RPC. The toughening mechanism of MWCNTs in RPC was also discussed. Moreover, the microstructures of the MWCNTs and cement hydration products were studied by scanning electron microscopy (SEM).

## 2. Materials and Methods 

### 2.1. Materials

Portland cement with a strength grade of 52.5 was used in the composite. Its chemical composition is provided in Table 1. Silica fume (Elkem 920U) with a specific area greater than 15 m^2^/g and SiO_2_ content greater than 85% was used. Blast furnace slag was also used as cementitious materials. Refined quartz sand with particle diameters in the range 0–0.3 mm was selected, and the particle size mainly distributed in the 0.15–0.3 mm range. Copper-plated steel fibers that were 12 mm long and 0.2 mm in diameter were employed, and its shape was straight. The tensile strength of the steel fiber was 2800 MPa. Polycarboxylic acid (SP) with a water-reducing rate greater than 35% was used as a water-reducing agent in the RPC mixes. 

**Figure 1:**
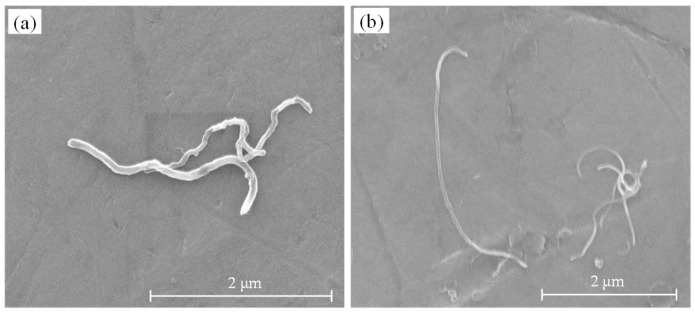
Scanning electron microscope image of type F (**a**) and type L (**b**) multi-walled carbon nanotubes.

The properties of MWCNTs used in this study are shown in Table 2. The micrograph of MWCNTs after dispersion is shown in Figure 1. Four different MWCNT contents (0.025, 0.05, 0.1, and 0.2 wt.%) were added into the RPC matrix. Note that the water used in the MWCNTs dispersion was included in the total amount of water.

### 2.2. Dispersion of Multi-Walled Carbon Nanotubes

The MWCNTs used in this experiment were produced using the chemical vapor deposition method. There was a large van der Waals force between the MWCNTs. A high efficiency nonionic surfactant (90% active contents, 68–70 °C cloud point) purchased from Chengdu Organic Chemicals (Chengdu, China) Co. Ltd. was used in this experiment. Its structure consisted of a hydrophilic group and an aromatic ring, which could improve the dispersibility of the MWCNTs significantly. Based on previous studies [22,23,24], MWCNTs powder and surfactant were placed in water and stirred evenly. Then, an ultrasonic disrupter (20 kHz, 450 W) was employed to disperse MWCNTs in water for 30 min. Finally, dispersion was subjected to centrifugation (2000 rpm, 30 min) and filtration treatment.

The dispersion contained 2 wt.% MWCNTs, which were uniformly black in color, and little sedimentation occurred due to the van der Waals forces after 90 days of static settling. The highly concentrated MWCNT-bearing liquid was diluted with water. Drops of this solution were placed on a sample holder and observed using an environmental SEM (scanning electron microscope, FEI Company, Hillsboro, OR, USA), as shown in Figure 1. Some of the MWCNTs were shortened during the dispersion process, which mainly occurred due to the ultrasonic vibration.

### 2.3. Preparation of Specimens

The mixture proportions are shown in Table 3. Firstly, cement, silica fume, and slag were dry-mixed for 2 min. Then, the fine sand was put into the mixing pot, followed by another 1 min of mixing. Secondly, the MWCNT dispersion, water and a superplasticizer were added to the mixed powders and mixed for 5 min. Finally, steel fiber was added into the mixture, and after a further 2 min of mixing the fibers were homogenously distributed throughout the fresh composite mortar. It should be noted that the mixing time affects the dispersibility of MWCNTs and steel fibers, so the mixing time of each group was fixed at 11 min to ensure the uniformity of the fiber distribution. The fresh RPC paste was cast into molds and compacted. According to GB/T 17671-1999 (China) [32], six cube samples (70.7 mm × 70.7 mm × 70.7 mm) and six flexural specimens (40 mm × 40 mm × 160 mm) were prepared for each group. The size of uniaxial direct-tension specimens was 15 mm × 50 mm × 300 mm. Twenty-four hours later, specimens were removed from the mold and cured under standard conditions (20 °C, 95% RH) until reaching the appropriate age for testing. 

### 2.4. Testing Procedures

In accordance with the GB/T 17671-1999 (China), the compressive strength of samples aged 28 days were tested at a loading rate of 0.4 mm/min using a 1000 kN Instron test platform. The flexural specimens were tested using a 250 kN Instron universal material test machine (Instron Asia Ltd., Shanghai, China) at a loading speed of 0.2 mm/min, and the distance between the two supports was 120 mm. After testing, some fragments were recovered using forceps and stored in acetone to prevent hydration of the specimen. Prior to analysis, the samples were put in an oven for 24 h. An FEI Quanta 650 FEG (FEI Company, Hillsboro, OR, USA) was used to investigate the microscopy of fractured cement composites. The surfaces of the RPC specimens were coated with a platinum/palladium layer to enhance their conductivity.

**Figure 2:**
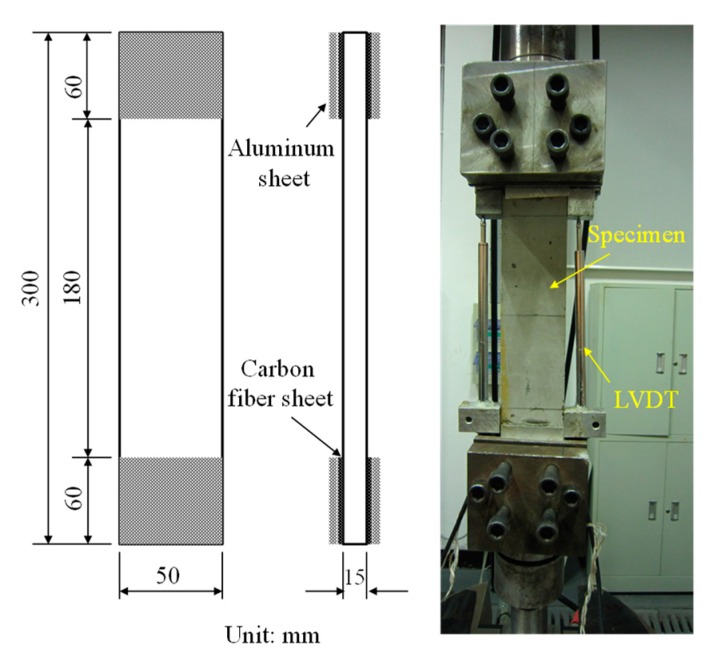
Uniaxial tensile specimen and test.

Uniaxial tensile test results were affected by the specimen size, machine stiffness and strain rate. The thickness of the specimen should be larger than the length of the reinforcing fibers to ensure that the fibers dispersed in the matrix uniformly. Previous studies showed that the cross-sectional area and the specimen length should not be too large to prevent tension eccentricity [33,34]. In this experiment, plate-type specimen with 50 mm × 15 mm cross section was used, and the length of the samples was 300 mm. Ball joints were installed at both ends of the holding device and were used to eliminate eccentricity produced during installation. Both ends of the specimen were polished, and an aluminum sheet was pasted on the polished surfaces by using epoxy resin. Tensile specimens were tested using a 250 kN Instron universal material test machine, and the loading rate for the tension testing was 0.1 mm/min. There were six specimens for each type of RPC. Linear variable differential transformer sensors (LVDT) were mounted on both sides of the specimen to measure tensile deformation, as shown in Figure 2.

## 3. Results

### 3.1. Compressive Test Results

**Figure 3:**
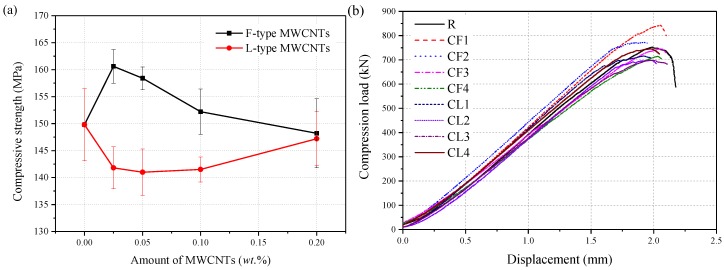
Compression test result (**a**) and typical compressive load-displacement curve (**b**).

The compressive test results showed the strength of each group varied from 141.0 to 160.6 MPa, as shown in Figure 3a. For the same matrix material, the cube compressive strength showed different degrees of enhancement at different MWCNT content. Compared with the control group, the compressive strength of the CF1 group was increased by 7.2%. With the addition of 0.05 wt.% and 0.1 wt.% F-MWCNTs, the compressive strength increased by 5.7% and 1.6%, respectively. However, when the F-MWCNT content increased to 0.2 wt.%, the compressive strength of the specimen decreased by 1.1%. For the composite containing 0.05 wt.% type-L MWCNTs, the compressive strength of RPC decreased by 5% compared with the control group. Compared with type-F MWCNTs, type-L MWCNTs with a high aspect ratio were more difficult to be dispersed equably. Re-agglomeration of type-L MWCNTs can be expressed as nanoscale holes or cracks that may cause stress concentrations and negatively impact the mechanical properties [19,20,21]. However, the effect of MWCNTs type on the compressive strength of RPC became insignificant gradually as the MWCNTs concentration increasing, and the strengths of CL4 and CF4 group were similar. 

In this study, the load-displacement curves for samples were tested using a 1000 kN Instron test platform, as shown in Figure 3b. The experimental results showed that the load-displacement curves of each group were similar, and the deformation capability of the RPC matrix had no significant change with the addition of MWCNTs. The matrix incorporating 0.025 wt.% F-MWCNTs exhibited the highest compressive strength.

### 3.2. Flexural Strength Results

**Figure 4:**
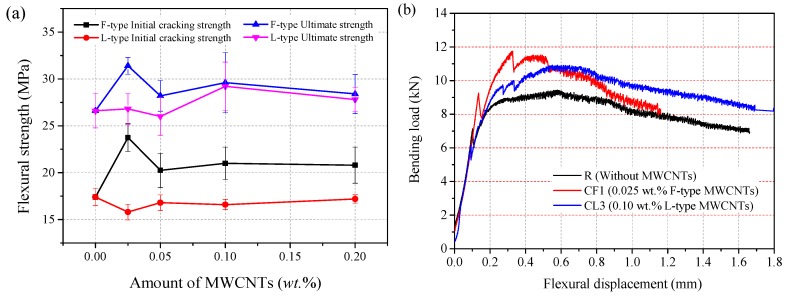
Flexural test results of each group (**a**) and typical load-displacement curve (**b**).

In the three-point bending test, six flexural specimens (40 mm × 40 mm × 160 mm) were prepared for each group. During the test, the mid-span displacement was recorded with LVDT. The flexural strength was calculated as

(1) $$\sigma =\frac{3PL}{2bd^{2}}$$

where *P* is the maximum load recorded during the test, *b* is the specimen width, and *d* is the specimen depth. Figure 4 gives the average flexural strength of each group. It was observed that, without any MWCNTs, the flexural strength of the R group had a maximum value of 26.6 MPa. Compared to the control group, the initial cracking strength and ultimate flexural strength of the CF1 specimen improved by 36.5% and 18%, respectively. Furthermore, the initial-cracking flexural strength of F-MWCNT-reinforced RPC composites with weight fractions of 0.05%, 0.1% and 0.2% showing a slight increase, up to 16.4%, 20.7%, and 19.5%, respectively. The ultimate flexural strength of the RPC with 0.1 wt.% L-MWCNTs increased by 2.6 MPa (or 9.8%), whereas the initial-cracking flexural strength decreased by 0.8 MPa (or –4.6%), compared with R group. The optimum content of F-MWCNTs and L-MWCNTs for samples was 0.025 wt.% and 0.1 wt.%, respectively.

Typical bending load versus displacement curves of specimens (R, CF1, and CL3) are shown in Figure 4b. The results showed that steel fibers, MWCNTs, and the matrix sustained the internal loads as a whole, up to the initial-cracking load. The initial cracking occurred in about 70–80% of the peak load, and initial-cracking flexural strength was determined by this critical point. After the curve reached the critical point, the steel fibers began to limit the development of macro-cracks. The steel fibers improved the ultimate strength because of high interfacial bond stress between the steel fiber and the matrix. Previous studies also showed that MWCNTs could delay the initiation of micro-cracking, which might result in the increasing of RPC initial-cracking strength [18,19,20,21,22,23].

### 3.3. Uniaxial Tensile Test Results

In this experiment, there were six specimens in each group for the tensile strength test. If the fracture occurred outside of the monitoring area, the test of that specimen was considered ineffective. The ultimate strain was the maximum strain at which the tensile load reached its peak value. Three or more valid data points were obtained for each group of specimens in this research, and the final measurement result was the average of these values. 

**Figure 5:**
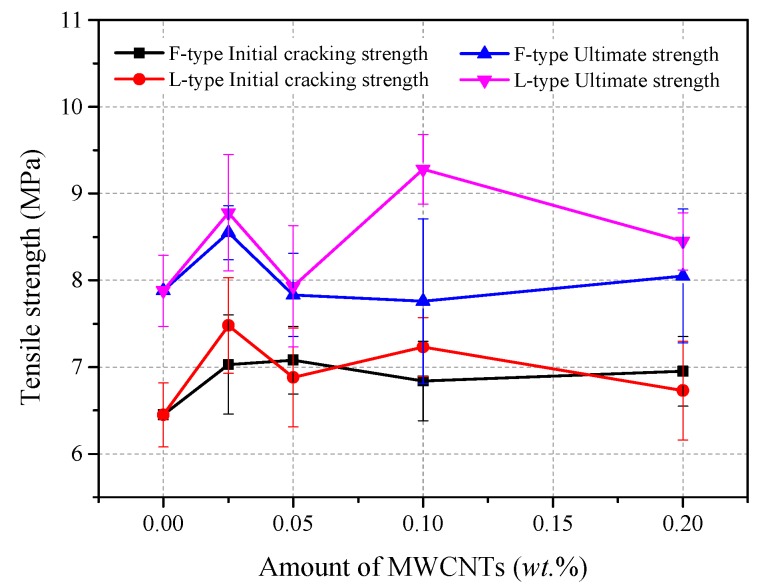
Direct tensile strength of each group.

**Figure 6:**
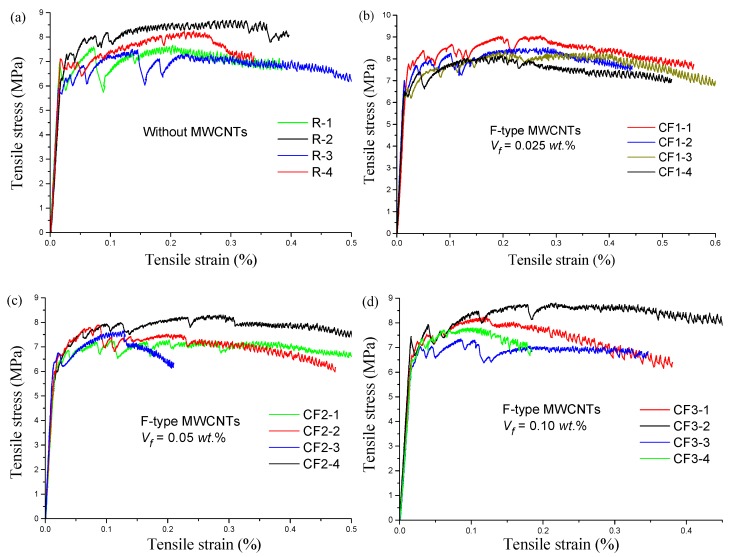

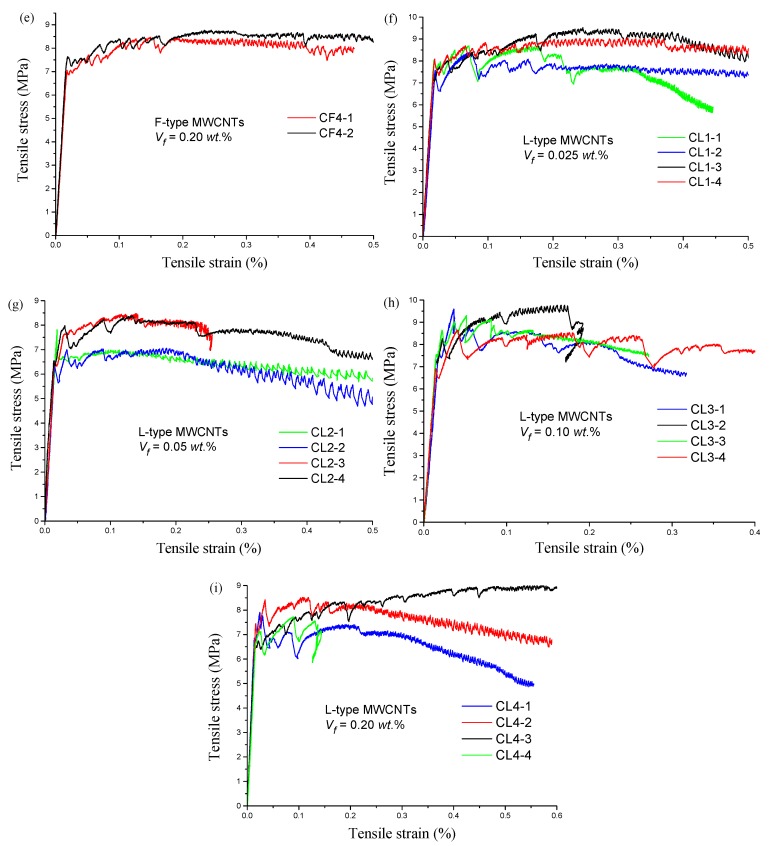
Uniaxial tensile stress–strain curves for each group: (**a**) R; (**b**) CF1; (**c**) CF2; (**d**) CF3; (**e**) CF4; (**f**) CL1; (**g**) CL2; (**h**) CL3; (**i**) CL4.

The direct tension test results are shown in Figure 5, and Figure 6 shows the direct–tension stress–strain curves of each group. From Figure 5, the initial cracking strength (*σ_in_*) and ultimate tensile strength (*σ_u_*) of the control group R were 6.5 MPa and 7.9 MPa, respectively. With the addition of MWCNTs, the initial cracking and ultimate tensile strengths of RPC improved significantly. For specimens with 0.025 wt.% F-MWCNTs, the initial cracking strength and ultimate tensile strength increased by 8.9% and 8.5%, respectively, and the ultimate strain reached 0.29%. Moreover, the initial cracking strengths of groups CF2, CF3, and CF4 were also enhanced to some degree. With the addition of 0.025 wt.% L-MWCNTs, the initial cracking strength and ultimate tensile strength increased by 16% and 11.4%, respectively. Uniaxial tensile stress–strain curves (Figure 6) showed that the initial cracking strain of each group was about 0.016%, and there was less differentiation between the specimens. However, the ultimate tensile strain of RPC with 0.025 wt.% L-MWCNTs and 0.2 wt.% F-MWCNTs was higher than other groups.

As shown in Figure 6, the tensile curves of the MWCNT/RPC composites could be divided into 3 stages: the elastic stage, the unstable strain-hardening, and the post-peak stage. In the elastic stage, there was a linear relationship between tensile stress and strain before the initial crack appeared. There was an abrupt reduction in the stress after initial matrix cracking, and then the tensile stress increased slowly with increasing strain. The brittleness of the RPC matrix diminished with the addition of steel fibers, and these randomly oriented fibers inhibited the propagation of cracks and increased the load-carrying capacity. When the load increased continuously, the number of crack branches increased, and the strain hardening was enhanced. After the peak load was reached, the RPC composites entered the strain-softening stage. The main cracks were apparent, and the crack width increased noticeably. With the high strength of the steel fibers, the failure mode of the specimen was the interfacial failure of the fiber and the matrix in the crack and pullout of the fiber. Multiple cracks spread throughout the specimen and most of the crack widths were lower than 50 μm before the load reached peak.

### 3.4. Toughness Index Based on Tensile Load–Displacement Curves

**Figure 7:**
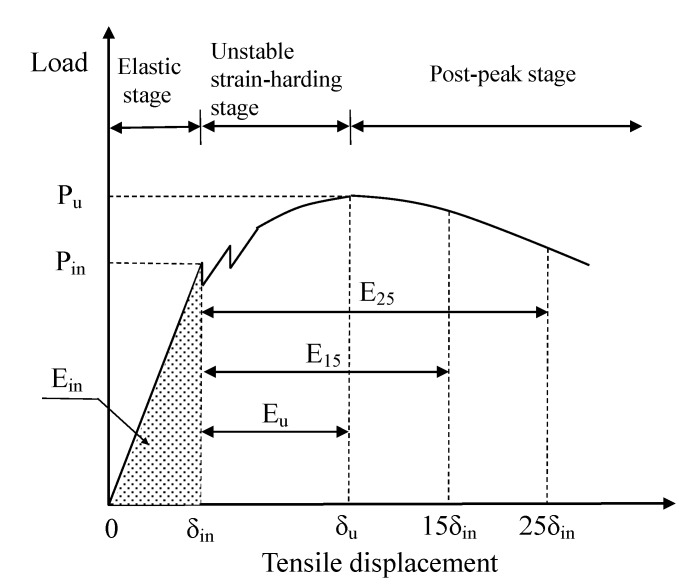
Illustration of determination of energy absorption capability of reactive powder concrete.

Tensile toughness characteristics and energy absorption capability significantly affects the durability and safety of structures built using RPC since a high strength often results in high brittleness. However, there is no widely accepted testing method for the characterization for the RPC toughness, and little information is presently available regarding the tensile toughness of RPC [35,36]. According to the existing toughness evaluation methods, the areas encircled by load–deformation curves between the initial crack point and a prescribed point were taken to determine the energy absorption capability of fiber-reinforced concrete [37,38]. In this research, tensile toughness parameters were also determined by using the areas under the tensile load–displacement curve. Figure 7 shows a typical tensile load–displacement curve of the RPC plate. In the elastic stage, the tensile load–displacement curves appear flat-and-straight and the load drops down sharply at the initial cracking load. The corresponding tensile load and displacement were defined as the initial cracking load P_in_ and the initial cracking deflection *δ_in_*. In the same way, *P_u_* and *δ_u_* were defined as the ultimate load and ultimate strength displacement, respectively.

To highlight the influence of MWCNTs on the energy absorption capability, we introduced the energy absorption factors that were obtained by dividing the area under the tensile load–displacement curve up to a specified displacement. For example, the shaded part of Figure 7, denoting *E_in_*, represents the energy that the RPC matrix absorbed at first cracking. The energy absorption capacity *E_u_* is equal to the area under the tensile load–displacement curve from *δ_in_* to *δ_u_*, so it can be expressed as follows:

(2) $$E_{u}=\int_{\delta_{\mathrm{in}}}^{\delta_{u}}P\left(\delta \right)d\delta$$

In order to describe the dissipated energy in the post-peak stage, the variables E_15_ and E_25_, defined as the energy absorption capacity after the peak load, were introduced as evaluation parameters. They were obtained by dividing the area up to a displacement of 15 and 25 times the initial crack displacement, respectively, by the area up to initial crack. *E_15_* and *E_25_* were calculated by Equations (3) and (4) as follows:

(3) $$E_{15}=\int_{\delta_{\mathrm{in}}}^{15\delta_{\mathrm{in}}}P\left(\delta \right)d\delta$$

(4) $$E_{25}=\int_{\delta_{\mathrm{in}}}^{25\delta_{\mathrm{in}}}P\left(\delta \right)d\delta$$

Table 4 lists the calculation results for the energy absorption capability of the RPC specimens with various MWCNT contents. Because of the brittleness of the RPC matrix, the value of *E_in_* was very low. The *E_in_* value of CF and CL groups were all significantly higher than that of the control group, which meant *E_in_* was sensitive to the MWCNTs content. The value *E_u_* for groups CF1, CF2, and CL1 significantly increased, but other groups showed no improvement. Two factors might be responsible for this situation: On the one hand, the ultimate strain decreased significantly with the increase in the MWCNT content, especially in the case of the CL group; on the other hand, the curve from the crack initiation to the peak-load was unstable, so it was difficult to measure *E_u_* accurately. *E_15_* and *E_25_* characterize the contribution of MWCNTs to improving the post-peak toughness of RPC. For specimens with 0.05 wt.% F-MWCNTs, the value of *E_u_* reached 2.367 N·m. compared to the R group, the value of *E_15_* and *E_25_* of CF1 group increased by 30.7% and 21.2%, respectively. It was worth noting that lower amounts of L-MWCNTs (0.025 wt.%) provided effective energy absorption capacity, while higher amounts (0.05 wt.%) of F-MWCNTs were required to achieve the same level of performance.

### 3.5. Scanning Electron Microscope Observations

**Figure 8:**
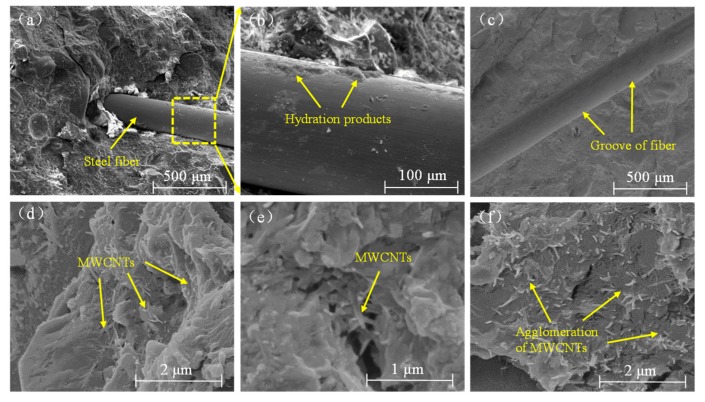
Scanning Electron Microscope analysis of steel fiber in the reactive powder concrete mortar (**a**–**c**), and multi-walled carbon nanotubes in the CF group (**d**,**e**) and CL group (**f**).

Because of the prominent effect by microstructure on mechanical capabilities, SEM was applied to analyze the micrograph of the MWCNTs-RPC specimens. Figure 8a shows a representative microstructure of the R group material. The main products of the zone were C-S-H gel and unhydrated portland clinker particles and cracks. Copper-coated steel fiber had a smooth surface, hence little hydration products of the cement could be seen on the surface of the fibers, as shown in Figure 8b. After pulling out the steel fiber, the groove on the surface of the hardened RPC was observed in Figure 8c.

As shown in Figure 8d, MWCNTs were well-embedded in the matrix and the hydration products were connected into a single region. As the arrow in Figure 8e indicates, the MWCNTs acted as a bridge across the pore or crack, and the ends of some of the MWCNTs were embedded in the matrix, while the other end was pulled out. However, the MWCNTs were not dispersed well in some regions of the CL4 sample. Figure 8f showed an area with a high concentration of MWCNTs. Due to the high surface energy, a high content of MWCNTs caused the aggregation of CNTs, and this resulted in the decline in the performance of RPC.

## 4. Discussion

Usually, the optimal CNT content in cement composites is influenced by the aspect ratios and dispersion method of the CNTs [14,39]. Compared with small aspect ratios of MWCNTs, high aspect ratios MWCNTs exhibited the same level of mechanical performance at lower concentrations, and they were found to be more difficult to disperse [19]. Meanwhile, excessive use of CNTs resulted in the significant reduction in the strength of composites [20,23,39]. From Figure 4 and Figure 5, the initial-cracking strength and ultimate strength of RPC was greatly improved with F-type MWCNTs. Compared with the control group, the initial cracking and ultimate tensile strength of CF1 specimens increased by 8.9% and 8.5%, respectively. With the same addition of L-MWCNT, the initial cracking and ultimate tensile strength improved 16.0% and 11.4% respectively. It demonstrated that MWCNTs with a higher aspect ratio could be enhanced when added MWCNTs were the same in quantity. Compared with the R group, the initial-cracking flexural strength of specimens with L-MWCNTs had no significant change. This might be related to the dispersion of MWCNTs, and L-type MWCNTs with high aspect ratio were found to be more difficult to disperse uniformly. However, the ultimate flexural strength of the RPC with 0.1 wt.% L-MWCNTs increased by 9.8%. It indicated that the addition of L-type MWCNTs had a positive effect on the crack resistance of RPC. As Figure 5 shows, the enhancement trend of L-MWCNTs decreased slightly with a further increase in the L-MWCNTs content. This may be attributed to the following reasons: first, the proposed method for dispersing MWCNTs in RPC was effective and the highest content of MWCNTs was only 0.2 wt.%; second, aggregated MWCNTs filled the voids between the large cement hydration products and acted as a crystal nucleus to speed up C–S–H formation in cement, which improved the microstructure of the composites. This explains why aggregated MWCNTs had a limited influence on mechanical properties at high concentrations.

**Figure 9:**
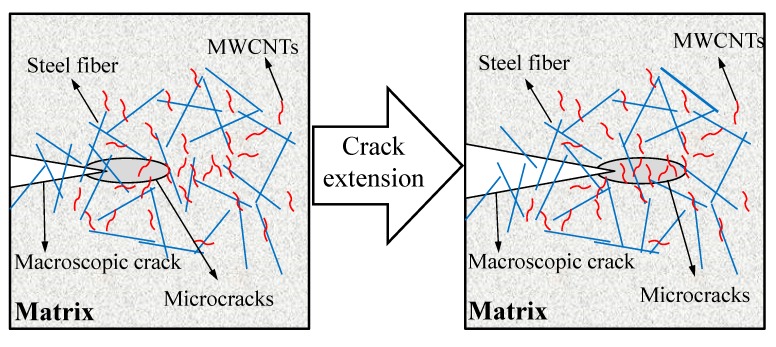
Schematic diagram of toughening mechanism of MWCNTs in RPC.

In this study, the hybrid fiber-reinforced RPC matrix containing nanofiber (MWCNTs) and high-modulus macrofiber (steel fiber) exhibited simultaneous improvement in the first crack strength, toughness, and ultimate tensile strength. For specimens containing 0.05 wt.% L-MWCNTs, the initial cracking strength and ultimate tensile strength of RPC were 7.2 MPa and 9.3 MPa, respectively. The reinforcement of the RPC matrix by steel fibers and MWCNTs, which provided continuity across the cracks, delays the initiation of propagation. In general, MWCNTs and steel fibers play different roles in the failure process: MWCNTs decelerate crack development from micro-cracks to short macro-cracks, and steel fibers restrict the development of macro-cracks, as shown in Figure 9. The fully dispersed MWCNTs were tightly wrapped by hydrated calcium silicate (C-S-H) gel, which improved the bonding strength between the MWCNTs and hydration products. It also filled the interstitial spaces in the hydration products, increased their density and effectively reduced the number of initial internal defects [40]. Therefore, MWCNTs could decelerate crack development at the nano scale and improve the initial cracking tensile strength of RPC [24]. After the initial cracking, the multiple-cracking behavior of RPC was mainly caused by the steel fiber pulling out. That explained why the RPC specimen incorporating 0.025 wt.% F-MWCNTs showed a better load-carrying capacity and tensile toughness.

## 5. Conclusions

In this study, the mechanical properties and microstructure of RPC blends containing two kinds of MWCNTs (type F and type L) were investigated. Test results showed that MWCNTs enhanced the compressive, flexural and tensile strength of RPC, and the addition of MWCNTs imparted strain-hardening and multiple-cracking behavior of RPC. Meanwhile, specimens containing L-MWCNTs had better tensile performance at lower CNT contents.

A toughness characterization method was proposed to establish an objective tensile toughness index for RPC. The effect of MWCNTs on the energy absorption was significant and the values of *E_in_*, *E_15_*, and *E_25_* were all higher than those for the control. The lower amounts of L-MWCNTs (0.025 wt.%) provided effective energy absorption capacity due to its high aspect ratios, while the higher amounts (0.05 wt.%) of F-MWCNTs were required to achieve the same level of mechanical performance.

The morphology analysis of the specimens demonstrated the mechanism for the improvement of the properties of RPC with the addition of MWCNTs. The MWCNTs connected the cement hydration products together and improved the microstructure of the RPC matrix. The pulling out and crack bridging of MWCNTs inhibited the growth of micro cracks and increased the toughness of the matrix.

## Figures and Tables

**Table 1:** Chemical properties of ordinary Portland cement.

Component	Fe_2_O_3_	Al_2_O_3_	CaO	MgO	SiO_2_	SO_3_	Loss
Amount (%)	2.91	4.58	61.08	3.19	19.50	2.50	3.60

**Table 2:** Properties of multi-walled carbon nanotubes.

Type	Length/μm	Aspect Ratios	Purity/%	Specific Surface Area/(m^2^·g^−1^)	Electric Conductivity/(s·cm^−1^)
F	2~5	40~100	98	300	30 × 10^−3^
L	5~10	250~500	98	300	30 × 10^−3^

**Table 3:** Multi-walled carbon nanotubes-reactive powder concrete composites mixture proportions.

Mix No.	Cementitious Materials (g)			Sand (g)	Water (g)	MWCNTs (wt.% of cement)	Steel Fiber (g)	SP (g)
	Cement	Silica Fume	Slag					
R	1000	250	200	1100	240	0	180	45
CF1	1000	250	200	1100	240	0.025	180	45
CF2	1000	250	200	1100	240	0.05	180	45
CF3	1000	250	200	1100	240	0.1	180	45
CF4	1000	250	200	1100	240	0.2	180	45
CL1	1000	250	200	1100	240	0.025	180	45
CL2	1000	250	200	1100	240	0.05	180	45
CL3	1000	250	200	1100	240	0.1	180	45
CL4	1000	250	200	1100	240	0.2	180	45

**Table 4:** Energy absorption capabilities of reactive powder concrete specimens with various multi-walled carbon nanotubes contents.

Sample	MWCNTs (wt.%)	*E_in_* (N·m)	*E_u_* (N·m)	*E_15_* (N·m)	*E_25_* (N·m)
R	0	0.057	1.656	1.689	2.855
CF1	0.025	0.068	2.404	2.018	3.517
CF2	0.05	0.065	2.367	2.207	3.461
CF3	0.1	0.059	1.430	1.771	2.786
CF4	0.2	0.064	1.794	2.063	3.516
CL1	0.025	0.07	2.201	2.24	3.591
CL2	0.05	0.062	1.498	1.838	2.962
CL3	0.1	0.061	1.238	2.013	2.838
CL4	0.2	0.062	0.884	1.856	3.167
